# STEPWISE – STructured lifestyle Education for People WIth SchizophrEnia: a study protocol for a randomised controlled trial

**DOI:** 10.1186/s13063-016-1572-1

**Published:** 2016-09-29

**Authors:** Rebecca Gossage-Worrall, Richard I. G. Holt, Katharine Barnard, Marian E. Carey, Melanie J. Davies, Chris Dickens, Yvonne Doherty, Charlotte Edwardson, Paul French, Fiona Gaughran, Kathryn Greenwood, Sridevi Kalidindi, Daniel Hind, Kamlesh Khunti, Paul McCrone, Jonathan Mitchell, John Pendlebury, Shanaya Rathod, David Shiers, Najma Siddiqi, Lizzie Swaby, Stephen Wright

**Affiliations:** 1 The University of Sheffield, Sheffield, UK; 2 Human Development and Health Academic Unit, University of Southampton Faculty of Medicine, Southampton, SO16 6YD UK; 3 Leicester Diabetes Centre, University Hospitals of Leicester NHS Trust, Leicester, UK; 4 Diabetes Research Centre, University of Leicester, Leicester, UK; 5 University of Exeter Medical School and NIHR Collaboration for Leadership in Applied Health Research and Care (CLAHRC) for the South West Peninsula, Exeter, UK; 6 University Hospitals of Leicester NHS Foundation Trust, Leicester, UK; 7 Greater Manchester West NHS Foundation Trust, Manchester, UK; 8 South London and Maudsley NHS Foundation Trust, London, UK; 9 R&D, Sussex Partnership NHS Foundation Trust; and, School of Psychology, University of Sussex, Brighton, UK; 10 Kings College London, London, UK; 11 Sheffield Health and Social Care NHS Foundation Trust, Sheffield, UK; 12 Southern Health NHS Foundation Trust, Southampton, UK; 13 Carer; former GP, North Staffordshire; Honorary Reader in the Division of Psychology and Mental Health at the University of Manchester, Manchester, UK; 14 Bradford District Care Trust, Bradford, UK; 15 Leeds and York Partnership NHS Foundation Trust, Leeds, UK

**Keywords:** Overweight, Obesity, Schizophrenia, First-episode psychosis, Antipsychotic medication, Behaviour change, Education, Lifestyle

## Abstract

**Background:**

People with schizophrenia are two to three times more likely to be overweight than the general population. The UK National Institute of Health and Care Excellence (NICE) recommends an annual physical health review with signposting to, or provision of, a lifestyle programme to address weight concerns and obesity. The purpose of this randomised controlled trial is to assess whether a group-based structured education programme can help people with schizophrenia to lose weight.

**Methods:**

Design: a randomised controlled trial of a group-based structured education programme.

Setting: 10 UK community mental health trusts.

Participants: 396 adults with schizophrenia, schizoaffective, or first-episode psychosis who are prescribed antipsychotic medication will be recruited. Participants will be overweight, obese or be concerned about their weight.

Intervention: participants will be randomised to either the intervention or treatment as usual (TAU). The intervention arm will receive TAU plus four 2.5-h weekly sessions of theory-based lifestyle structured group education, with maintenance contact every 2 weeks and ‘booster’ sessions every 3 months. All participants will receive standardised written information about healthy eating, physical activity, alcohol and smoking.

Outcomes: the primary outcome is weight (kg) change at 1 year post randomisation. Secondary outcomes, which will be assessed at 3 and 12 months, include: the proportion of participants who maintained or reduced their weight; waist circumference; body mass index; objectively measured physical activity (wrist accelerometer); self-reported diet; blood pressure; fasting plasma glucose, lipid profile and HbA_1c_ (baseline and 1 year only); health-related quality of life (EQ-5D-5L and RAND SF-36); (adapted) brief illness perception questionnaire; the Brief Psychiatric Rating Scale; the Client Service Receipt Inventory; medication use; smoking status; adverse events; depression symptoms (Patient Health Questionnaire-9); use of weight-loss programmes; and session feedback (intervention only). Outcome assessors will be blind to trial group allocation.

Qualitative interviews with a subsample of facilitators and invention-arm participants will provide data on intervention feasibility and acceptability. Assessment of intervention fidelity will also be performed.

**Discussion:**

The STEPWISE trial will provide evidence for the clinical and cost-effectiveness of a tailored intervention, which, if successful, could be implemented rapidly in the NHS.

**Trial registration:**

ISRCTN19447796, registered on 20 March 2014.

**Electronic supplementary material:**

The online version of this article (doi:10.1186/s13063-016-1572-1) contains supplementary material, which is available to authorized users.

## Background

Schizophrenia is a psychotic illness that affects approximately 1 % of the population. Mortality rates are increased two- to four-fold in people with schizophrenia and life expectancy is reduced by 10–20 years [1–3]. Approximately 75 % of all deaths in people with schizophrenia are caused by physical illness with cardiovascular disease being the commonest cause of death [1]. The prevalence of type 2 diabetes is increased two-fold in people with schizophrenia [4]. Overweight and obesity contribute to this excess morbidity and mortality. Recent studies indicate that obesity is two to three times more prevalent among people with schizophrenia than in the general population and this occurs early in its natural history [5].

The rates of overweight and obesity have increased substantially over the last three decades in people with schizophrenia and to a much greater extent than in the general population [6]. Reasons for this increase of obesity are complex and relate to environmental factors, such as poor diet and physical inactivity, as well as disease and treatment effects. Weight gain is a common adverse effect of antipsychotic medication, affecting between 15 and 72 % of patients [7]. Most weight gain occurs early in treatment with between 37 and 86 % of those experiencing a first episode of psychosis also experiencing more than 7 % weight gain in 12 months [8], often occurring within 12 weeks of treatment initiation [9]. Longer-term observational studies suggests that weight gain continues for at least 4 years albeit at a slower rate [10].

Individuals with schizophrenia are more likely to consume a diet that is rich in fat and refined carbohydrates while containing less fibre, fruit and vegetables than that of the general population [11–13]. Physical inactivity and the social and urban deprivation experienced by many people with schizophrenia may contribute further to their increased obesity rates [12–15]. These factors suggest that it may be possible to address the problem of obesity through appropriate lifestyle intervention.

A meta-analysis of nonpharmacological interventions in people with schizophrenia [16] has shown that these led to a mean reduction in weight of 3.12 kg over a period of 8–24 weeks. There were commensurate reductions in waist circumference and improvements in cardiovascular risk factors. The benefits of the programmes were seen irrespective of the duration of treatment, whether the intervention was delivered to an individual or in a group setting, whether the intervention was based on cognitive behavioural therapy or a nutritional intervention or whether it was designed to promote weight loss or prevent weight gain. There was overlap across intervention type, such that most cognitive behavioural therapy programmes also included a diet- or exercise-based intervention. Outpatient interventions appeared more effective than inpatient settings.

The meta-analysis, however, acknowledges a number of limitations of the trials, including small sample sizes and the lack of long-term follow-up. Most previous studies do not extend beyond 12 weeks and hence the impact of the interventions in the longer term remains unknown. The few studies reporting long-term effects suggest that these may persist after the end of the programme for up to 1 year but others suggest that long-term behaviour change is difficult to achieve [17]. The meta-analysis called for longer trials with larger numbers, with a focus on weight maintenance after the initial intervention.

The majority of evaluated weight-loss interventions, including diabetes prevention programmes, have utilised intensive one-to-one counselling strategies to promote behaviour change; however, these are challenging for direct implementation within current UK community care settings because of resource limitations [18]. Structured education is an alternative to one-to-one counselling and refers to group-based, patient-centred educational programmes that have a clear philosophy; have a written curriculum that is underpinned by appropriate learning and health behaviour theories; are evidence-based; and are delivered by trained, quality-assessed, educators [19]. Structured education has been widely advocated in England as a potentially cost-effective method of promoting self-management and behaviour change in individuals with chronic disease. Importantly, this approach has recently been adopted in the promotion of lifestyle change by the National Institute for Health and Care Excellence (NICE) diabetes prevention guidance [20].

The STEPWISE intervention is based on the diabetes prevention programme ‘Let’s Prevent Type 2 Diabetes’ [21] (often abbreviated to ‘Let’s Prevent’), which was developed by the Diabetes Education and Self-management for Ongoing and Newly Diagnosed (DESMOND) team.

DESMOND was the first nationally established, structured education programme and was originally designed for people with type 2 diabetes. The programme was effective at promoting lifestyle change, including weight loss, and reducing symptoms of depression in a multicentred randomised controlled trial (RCT) [22]. Let’s Prevent [23] is 6 h long and is designed to promote increased physical activity (specifically walking), a healthy diet through reduced fat and saturated fat intake and increased fibre, and weight loss by enabling participants to self-regulate their behaviour actively using self-monitoring (feedback), relapse prevention (identifying and addressing barriers to change) and goal-setting strategies. It also includes standardised educator/facilitator training and a quality assurance programme.

Given the rapid weight gain experienced by people with a first episode of psychosis [7], there is an urgent need to develop interventions that lead to long-term reductions in overweight and obesity. There is also a paucity of data in people with first-episode psychosis and so this group is included in the STEPWISE trial.

## Aims and objectives

The aim of the STEPWISE trial is to evaluate the extent to which a structured lifestyle education programme, when delivered to adults with schizophrenia, including those with schizoaffective disorder or first-episode psychosis, in a community mental health setting, can support weight loss at 1 year.

Having adapted the DESMOND ‘Lets Prevent’ intervention for people with schizophrenia, schizoaffective disorder or first-episode psychosis and their health care professionals [24], the specific objectives of the trial are:

1. To undertake a multicentre RCT to test the hypothesis that a structured self-management lifestyle education programme can lead to a significant increase in physical activity and improved diet leading to clinically relevant weight loss after 1 year
2. To ensure fidelity of the intervention when scaled up, through a robust assessment of its delivery
3. Through qualitative research, to assess whether the intervention, when scaled up, is appropriate for and acceptable to mental health services and people with schizophrenia, schizoaffective disorder or first-episode psychosis
4. To undertake an economic evaluation of the intervention
5. To develop a quality assurance framework for facilitators delivering the intervention

## Methods

**Fig. 1:**
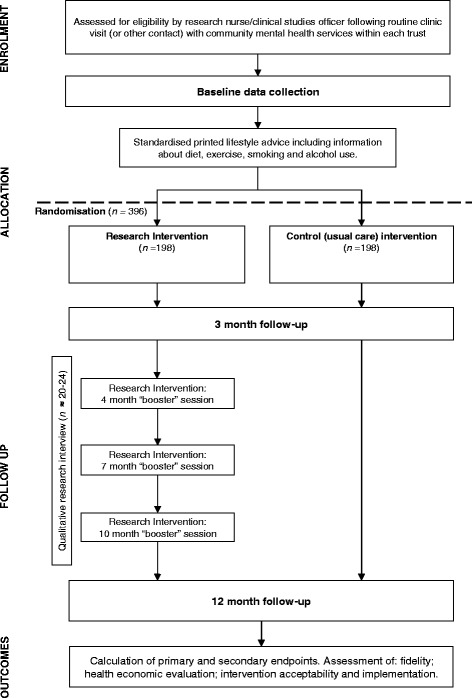
Trial design

The trial is a multicentre, two-arm, parallel-group RCT of the STEPWISE structured lifestyle education programme. The trial design is summarised in Fig. 1. The study was planned and implemented in concordance with the Consolidated Standards of Reporting Trials (CONSORT) [25]. Public and Patient Involvement was actively sought in the development of the trial and will continue throughout the trial.

### Setting

The study will take place within community mental health teams (CMHTs), including early intervention services, in 10 mental health NHS trusts in the rural and urban locations of Sheffield, Bradford, Leeds and York, Greater Manchester, Somerset, Devon, Cornwall, Sussex, Hampshire and South London.

### Participants

Adults are eligible for inclusion in the study if they:

1. Are aged 18 years or older
2. Have a diagnosis of schizophrenia or schizoaffective disorder (defined *by International Classification of Diseases, version 10* (ICD-10) codes F20, F25) or first-episode psychosis (defined as less than 3 years since presentation to the mental health team) using case note review
3. Are being treated with antipsychotic medication; for those with schizophrenia or schizoaffective disorder, the treatment duration should be at least 1 month prior to entry into the trial
4. Are able to give written informed consent
5. Are able and willing to attend and participate in a group education programme
6. Are able to speak and read English
7. Have a Body Mass Index (BMI) ≥25 kg/m^2^ or are concerned about their weight. For participants from South Asian and Chinese backgrounds, the BMI threshold is reduced to ≥23 kg/m^2^

People are excluded from the study if they have a:

1. Physical illness that could seriously reduce their life expectancy or ability to participate in the trial
2. A coexisting physical health problem that would, in the opinion of the principal investigator, independently impact on metabolic measures
3. Mental illness that could seriously reduce their ability to participate in the trial
4. Current pregnancy or are less than 6 months postpartum
5. A condition associated with significant weight gain, e.g. Cushing’s syndrome
6. Significant alcohol or substance misuse, which, in the opinion of the principal investigator, would limit the patient’s ability to participate in the trial
7. A diagnosis or tentative diagnosis of psychotic depression or mania
8. A primary diagnosis of learning disability

People will also be excluded if they are currently (or within the past 3 months) engaged in a systematic weight management programme.

### Selection and randomisation

The trial will be promoted within clinical teams and in areas where community mental health services are delivered. Members of the research team will work with clinical teams within the mental health trust to identify potentially eligible patients from their caseload. Patients can also self-refer, using information displayed on posters or leaflets, if they are interested in taking part.

After baseline assessments, participants will be randomised using the Sheffield Clinical Trials Research Unit’s (CTRU) remote randomisation system. A randomisation list generated using permuted blocks of random sizes will be used to allocate the participants to either treatment as usual (TAU) plus the STEPWISE lifestyle education programme or TAU alone in a 1:1 ratio, stratified by site and time since the start of antipsychotic medication (up to 3 months or longer than 3 months). Where the exact time is unknown, an approximate duration will be acceptable for the purposes of randomisation.

A member of the site team will inform the participants of their allocation. Outcome assessors will be blind to treatment allocation. Blind (or suspected) breaks will be recorded on the Case Report Form and reported periodically to the trial oversight committees. Due to the nature of the intervention participants cannot be blinded.

### Research intervention

#### Precourse information

Participants allocated to the research intervention will be contacted by the session coordinator (administrator) and provided with precourse information (e.g. an introductory letter and leaflet), which will confirm when the sessions will take place and what to expect.

#### STEPWISE education programme

The intervention lasts for approximately 12 months. Participants allocated to the intervention will receive a foundation course of four weekly 2.5-h (including breaks) group sessions delivered by two trained facilitators. The group sessions will involve approximately six to eight participants, although this may vary due to recruitment and attrition. The foundation course will be followed by 1:1 support contact lasting about 10 min, approximately every 2 weeks for the remainder of the intervention period, from a facilitator in order to support behaviour change. Ideally, support contact will be personalised and carried out face-to-face or by telephone. Participants will be invited to attend group-based booster education sessions at 4, 7 and 10 months post randomisation. Group attendance and receipt of support contact will be recorded.

Intervention sessions include: individual personal stories; taking control of weight; healthier food and drink choices; the relationship between weight and medication; and the relationship between calories and portions and physical activity (see Additional file 1). The intervention has a written curriculum to ensure consistency and resources that are in line with meeting its person-centred philosophy. Participants will not be ‘taught’ in a formal way, but rather supported to discover and work out knowledge for themselves, and to allow this to inform their goals and plans.

Participants attending education sessions will be invited by facilitators to complete a ‘Session Feedback’ form at the end of each session. The aim is to capture a self-report of empowerment and health belief during the course; the facilitators will not read the forms. Participants are not encouraged to bring someone with them to the intervention sessions; however, if they do, this will be documented.

#### Facilitator training

At least four health care professionals or associated staff at each location will receive facilitator training to deliver the STEPWISE education programme. Training comprises: the core DESMOND philosophy (1 day); and, specific content and delivery of the STEPWISE programme including content and delivery of booster sessions and support contact (3 days). Training will be preceded by a set of preparation exercises. Training will follow the philosophy and psychological principles that underpin patient education initiatives.

### Control arm

Participants in the control arm will receive TAU. There is significant variability in the provision of physical health care, despite the NICE guidelines on treatment and management of schizophrenia regarding healthy eating and physical health [26].

In order to standardise usual care in both groups, as far as possible, centres will provide printed advice to all participants at baseline (prior to randomisation) on the risk of weight gain and lifestyle advice, including information about diet and physical activity, and smoking and alcohol use (as appropriate). We will record any uptake of weight management and/or physical activity programmes by all participants during the study, and collate information from centres about uptake of NICE recommendations more widely.

### Outcome measures

Consistent with the primary objective of this trial, the primary endpoint is change in weight (kg) at 12 months after randomisation.

**Fig. 2:**
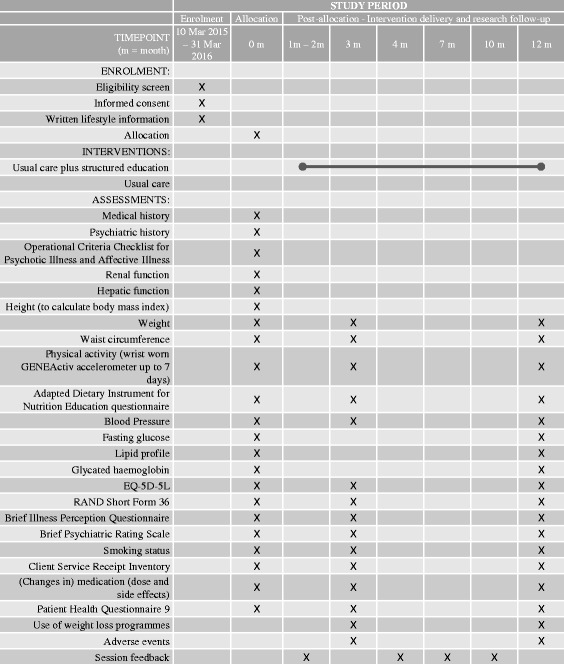
SPIRIT Figure – Schedule of enrolment, interventions, and assessments, based on recommendations for Interventional Trials (SPIRIT) figure, for the STEPWISE trial. Following enrolment, all participants receive written lifestyle information; and, before collection of 0 month assessments. Participants are then randomised to either usual care with structured education or usual care. Participants allocated to structured education are invited to: a) 4 weekly 2.5 hour group sessions (delivered by trained facilitators); group booster sessions at 4, 7 and 10 months post-randomisation; and, 1:1 support contact between month 2 and month 12. Research assessments are completed with all participants at 0, 3 and 12 months (as indicated)

Secondary outcomes include: proportion of participants who maintained or reduced their weight, waist circumference, body mass index, physical activity captured via wrist accelerometer, blood pressure, fasting plasma glucose, lipid profile and HbA_1c_; behaviour change (self-reported diet; smoking status) and psychosocial factors including quality of life (RAND SF-36 [27]and EQ-5D-5L [28]); health beliefs (adapted Brief Illness Perception Questionnaire [29]; Brief Psychiatric Rating Scale [30]) and cost-effectiveness (Client Service Receipt Inventory [31]). Secondary outcomes also include: medication use; adverse events; depression symptoms (Patient Health Questionnaire-9 [32]); use of weight-loss programmes; and session feedback (intervention participants only). All outcome measures will be assessed at baseline and after 3 and 12 months (except where stated) to measure if there is an effect and, if so, whether this is sustained in the longer term. Follow-up windows for 3- and 12-month follow-ups will be defined as minus 2 weeks and plus 4 weeks to allow time for missed appointments. A schedule of enrolment, interventions, and assessments based on the SPIRIT 2013 Figure [33] is shown in Fig. 2.

The research nurse or clinical studies officer will administer all questionnaires and other assessments either at the participant’s home or in the NHS trust. Additional information (e.g. medication) may be obtained from the patient’s notes. All participants will receive a £20 voucher at the 12-month assessment.

The operational criteria OPCRIT Checklist for psychotic and affective illness will be completed from case note review within 10 weeks post baseline visit to provide baseline characteristics. Collection of fasting blood sample and accelerometer data will be permitted after randomisation where recruitment is close to the scheduled education course.

#### Safety assessments

There are few anticipated adverse effects of this structured lifestyle intervention. The development of the intervention ensured that it was tailored to the needs of people with schizophrenia, schizoaffective disorder and first-episode psychosis. There is a risk that anxiety about weight and its complications may be increased. If the intervention is unsuccessful this may lead to feelings of poor self-esteem. These risks probably are outweighed by the risk of widening health inequality and worsening health among people with schizophrenia if the intervention is not assessed.

The following are expected serious adverse events for the patient population:

1. Psychiatric hospitalisation
2. Self-harm
3. Suicide attempt
4. Death from suicide

Adverse events will be monitored at 3- and 12-month follow-ups. All serious adverse events that are also both ‘unexpected’ (that is, the type of event is not listed in the protocol as an expected occurrence); and ‘related’ (that is, it resulted from administration of any of the research procedures) will be reported by sites to the central team and the sponsor for expedited reporting to the trial oversight committees and the Research Ethics Committee.

### Sample size

The sample size calculation is based on data obtained from two sources, both of which evaluated behavioural interventions for weight loss in people prescribed antipsychotic medication for schizophrenia. Das et al. undertook a systematic review of randomised and nonrandomised controlled trials which reported between-group differences of 1.5 to 6 kg with standard deviations of around 5 kg [34]. The second source was data on overweight and obese UK patients with severe mental illness in which 51 people with schizophrenia were followed up for at least 1 year; among these, the weight change was 7.7 kg with a standard deviation of 6.5 kg [35]. We propose to detect a difference of 4.5 kg, which is both clinically meaningful (being on average around a 5 % reduction in body weight) [36] and appears compatible based on previous work. Assuming a conservative estimate of the standard deviation (SD) of 10 kg, 95 % study power, and two-sided significance level of 5 %, 130 participants per arm (260 in total) are required in order to detect a minimum clinically important difference of 4.5 kg.

Since the intervention is delivered in groups, the outcomes of the participants within the same group may be correlated. Assuming on average of seven participants per group, and an intraclass correlation of 5 % in the intervention arm, the sample size will be inflated by a design effect of 1.3 in the intervention arm in order to allow for this, which yields revised sample sizes of 169 and 130 in the intervention and control arms, respectively (299 in total). To maintain a 1:1 allocation, 158 participants per arm are required to reproduce this power. We further anticipate a conservative dropout rate of around 20 % (higher than that observed in similar studies [37], giving a final total of 198 participants per arm. With 10 centres, this requires 40–50 participants per centre (rounded upwards), 20–25 of whom will receive the intervention in three to four groups.

### Internal pilot

The trial includes an internal pilot which will assess whether it is feasible to recruit and retain sufficient participants. By 11 September 2015, the middle of the project (month 24):

1. Ten centres should have been initiated and should have recruited their first participant
2. Two hundred and fifty participants should have been recruited
3. Six centres should have completed their first STEPWISE course
4. Ninety-six participants should have been followed up to their 3-month outcome assessment

The trial will be considered infeasible and will be stopped if one or more of the following conditions apply:

1. Fewer than six centres have recruited their first participant
2. Fewer than 125 participants have been consented
3. Fewer than three centres have completed their first 4-week STEPWISE course
4. Fewer than 75 % of those followed up to their 3-month outcome assessment have contributed valid weight (primary outcome at 12 months) data at this time point

### Data analysis

Data will be analysed and reported according to the guidelines of the revised CONSORT statement for randomised controlled trials [25].

The analysis will be performed on an intention-to-treat basis and all statistical tests will be two-tailed at 5 % significance level. Demographics and baseline characteristics will be summarised and assessed for comparability between the intervention and control arms [38, 39].

The primary outcome will be assessed by fitting a marginal generalised estimating equation model (GEE) adjusted for baseline weight, using robust standard errors and an exchangeable correlation structure. This model incorporates an adjustment for potential clustering or correlation among the outcomes of participants treated in the same group. A 95 % confidence interval for the difference in weight between the lifestyle intervention and control arms will be reported with its associated *P* value. A sensitivity analysis will be performed in the same manner, which will include baseline covariates and any observed imbalances. In case of missing data, the missing data mechanism will be explored and multiple imputation approach applied to assess the robustness of the findings.

Of key interest is whether the intervention conveys the same effect among recently diagnosed patients (i.e. patients experiencing first-episode psychosis) as it does among those established on antipsychotic medication for a greater period of time. We will investigate this in the analysis by fitting an interaction term between treatment group and the time since starting antipsychotic medicine (including a nonlinear term if appropriate). The treatment effect will be presented graphically for different subgroups of the time elapsed since commencing medication.

Other continuous outcomes will be analysed and reported in the same manner as the primary outcome. Analysis of binary outcomes will be undertaken using a marginal generalised logistic linear regression model within the GEE framework and difference between treatment groups will be reported as odds ratios with associated 95 % confidence intervals and *P* values.

### Economic evaluation

The economic evaluation will be from a health and social care and societal perspective. The number of intervention sessions received will be centrally recorded. The cost of the intervention will be based on staff time plus overheads (capital and administrative) and an element for training and supervision. Other service use will be recorded at baseline and at 3- and 12-month follow-ups using the interviewer-administered Client Service Receipt Inventory, and will include:

- primary care
- secondary care, including inpatient costs (specialist mental health and physical health services)
- social care
- psychotropic and other medication, and
- informal care from families or friends (expressed in terms of hours per week spent on specific tasks because of the participant’s health problems)

Service costs will be calculated by combining the above data with appropriate unit cost information [40, 41]. Informal care time will be valued using average wage rates with sensitivity analyses using minimum wage rates and the value of a homecare worker. Lost work time will also be recorded for those in employment and valued using average wage rates. Total health and social care costs and societal (i.e. including informal care and lost employment) costs will be reported and compared between the two groups for the 1-year follow-up. A regression model will be used with baseline costs controlled for and bootstrapped 95 % confidence intervals generated given the expected skewed cost distribution.

Cost-effectiveness will be assessed by combining the cost data (from both perspectives) with the primary outcome measure and QALYs. The latter will be generated from the EuroQol five dimensions, five levels questionnaire (EQ-5D-5L) using UK tariffs and area under the curve methods. If the intervention results in lower (higher) costs and better (worse) outcomes then it will be dominant (dominated). In the event of higher costs and better outcomes (or lower costs and worse outcomes), incremental cost-effectiveness ratios (ICERs) will be constructed to show the cost per extra unit of weight loss or extra QALY gained. Uncertainty around the ICERs will be addressed by constructing cost-effectiveness planes using 1000 bootstrapped cost-outcome pairs and cost-effectiveness acceptability curves (CEACs). The latter will indicate the probability that the intervention is more cost-effective than TAU for different threshold values placed on a unit reduction in weight or one more QALY gained. The NICE uses a threshold of £20,000–30,000 for a QALY gain and so the CEACs will include this value in the range of values. There are no recognised threshold values of a unit reduction in weight and so values will be reported at which the intervention or TAU has a 50 %, 70 %, 80 % and 90 % likelihood of being the most cost-effective option.

Sensitivity analyses will be conducted by varying the costs of the intervention, informal care and lost employment. QALYs based on the SF6D (derived from the RAND Short Form 36-item Health Survey; SF-36) will also be used in sensitivity analyses given that this arguably has better distributional properties than EQ-5D-5L-based QALYs in this patient population [42]. The EQ-5D-5L has a clear ceiling effect in people with schizophrenia while the SF6D is normally distributed.

### Process evaluation

A process evaluation will be undertaken ‘to explain discrepancies between expected and observed outcomes, to understand how context influences outcomes, and to provide insights to aid implementation’ [43]. We will use a modified version of Linnan and Steckler’s framework for process evaluation [44]. The context for intervention implementation will be explored using qualitative interviews, schedules for which will be developed by a multidisciplinary team including potential participants and will investigate whether the intervention, when scaled up, is appropriate for and acceptable to mental health services (i.e. facilitators) and service users. We will explore other aspects (e.g. reach, dose delivered and received) quantitatively.

The methods of recruitment will also be explored both qualitatively and quantitatively. We will not produce a composite score for implementation as proposed by Linnan and Steckler, as we do not believe such a score would have validity or utility for our intended audience.

### Qualitative component of STEPWISE trial

#### Participant interviews

Approximately 20–24 participants will be purposively sampled, from those consenting to be contacted about the qualitative interview, to recruit different genders and ages.

All interviews will be via telephone or face-to-face and last about 1 h depending on the participant’s response and wishes. Interviews will not be conducted until after the 3-month follow-up visit. The qualitative research will also inform the quantitative aspects of the process evaluation.

A semistructured interview topic guide, will contain general open questions exploring the experience and acceptability of the intervention to participants. It will also include questions intended to elicit themes outlined in the existing published literature such as the barriers to, and facilitators of, the use of lifestyle interventions in people with schizophrenia [45–47].

#### Facilitators interviews

The process evaluation will explore health care professionals’ views on the experience of incorporating the lifestyle intervention into practice through individual semistructured interviews with approximately 20 facilitators.

May’s ‘Normalisation Process Theory’ (NPT) will be used as a theoretical framework to understand better the conditions necessary to support the introduction, embedding and integration of protocolised lifestyle interventions as routine elements of care [48]. While NPT emphasises the influence of social systems on behaviour, the study will also utilise relevant domains from the Theoretical Domains Framework (TDF) [49, 50] which emphasises individual influences on behaviour. We will also collect facilitator characteristics (e.g. level of education). The process evaluation will inform the interpretation of the trial results and subsequent policy-making, in line with the MRC’s Complex Intervention Framework [43].

All semistructured interviews will be audio-taped and fully transcribed using the National Centre for Social Research ‘Framework’ approach [51].

#### Facilitator training and assessment of intervention fidelity

A fidelity assessment will be undertaken to investigate whether the intervention can be delivered faithfully when scaled up. As the facilitators’ delivery of the intervention is key, an assessment of facilitator performance in each of the participating centres will be conducted during the RCT.

The facilitator training programme and intervention fidelity checks will be undertaken through the DESMOND collaborative and based on the standardised and established criteria currently used to provide quality assurance in the DESMOND National Programme.

During the trial, the delivery of individual facilitators at each centre will be assessed against criteria derived directly from the STEPWISE programme. This will enable the study team to identify if there is any deviation from the delivery taught in training, and will also provide evidence for establishing a benchmark for delivery and facilitator competencies when the intervention is scaled up. All training and assessment procedures will be supportive and transparent.

Although it is not possible to identify the final suite of intervention fidelity tools, these are likely to include assessment of content delivery; assessment of facilitator behaviour against a set of criteria derived from the STEPWISE programme itself; and assessment of facilitator talk time, using the DESMOND Observational Tool (DOT). The latter assessment involves an independent assessor scoring activity in the session (e.g. who is speaking; miscellaneous activity) through use of a 10-s audio prompt (audible only to the assessor). It has been shown that the amount of time facilitators or participants spend speaking is predictive of the changes observed in the participants’ illness perceptions: less facilitator talk leads to a greater change in participants’ beliefs about their condition [36] and is a precursor to behaviour change. Intervention fidelity assessments will be used to inform the outcomes of the trial.

### Trial supervision

Sheffield Health and Social Care NHS Foundation Trust will act as the sponsor for the trial and, therefore, will have overall responsibility for the trial along with the chief investigator. Trial management will be provided by the Sheffield Clinical Trials Research Unit (CTRU), which adheres to its Standard Operating Procedures. The study will use the CTRU’s in-house, access-controlled, data management system for the capture and storage of participant data. Level and frequency of monitoring has been agreed with the sponsor. Three committees will govern the conduct of this study: the Trial Steering Committee (TSC); the Data Monitoring and Ethics Committee (DMEC); and the Trial Management Group (TMG). The TMG will oversee the day-to-day management of the trial. The DMEC is the only group with access to the unblinded outcome date while the trial is underway.

## Discussion

Several previous RCTs [16, 17] and long-term observational studies [35] have shown that lifestyle interventions are effective among people with schizophrenia. However, the current RCT data are limited because of the small numbers of participants involved and their short duration. In addition, few studies have included people with a first episode of psychosis and yet weight gain occurs commonly and rapidly in this group of individuals after treatment initiation. This trial will address these limitations by recruiting a large number of participants who will be followed for a year. We have also specifically included people with first-episode psychosis, where the aim of the intervention may be the prevention of weight gain rather than weight loss.

The importance of managing obesity and overweight in people with schizophrenia is recognised in a number of national and international guidelines. These advocate regular assessment of weight and the implementation of lifestyle modification to address weight gain. Despite the NICE guidance that recommends provision of a combined healthy eating and physical activity programme by mental health care providers, there is uncertainty about how to implement this, reflected by the large variation in the provision of care between different NHS trusts. This trial is being undertaken in 10 mental health trusts from differing geographical areas in England, serving different populations, to maximise generalisability of the findings. If this trial shows the STEPWISE intervention to be effective, the diversity of centres in the study should aid the implementation of the intervention more widely across the NHS.

The intervention is based on the existing Let’s Prevent programme of the DESMOND collaborative. The DESMOND programme is approved by the NICE for people with, or at risk of, type 2 diabetes. It has a well-established training and evaluation process, which could be extrapolated to mental health care professionals wishing to implement STEPWISE.

Although lifestyle programmes have been introduced in some mental health settings, no research has been performed to determine the acceptability of these programmes. People with severe mental illness have similar levels of interest in their cardiovascular health as the general population but are not always able to prioritise their physical health [52]. It is important to assess the acceptability of lifestyle programmes for patients and mental health care professionals. This trial will provide information about this issue.

Treating obesity with either lifestyle or pharmacological interventions is cost-effective in the general population but it is important to evaluate whether this is true for people with schizophrenia. The DESMOND programme has been shown to be cost-effective; using 2010 real-world costs of the intervention, the lifetime incremental cost was £82 and mean incremental cost per QALY was £2113 [53].

By 11 September 2015 the trial had met (and in some instances substantially exceeded) feasibility criteria included in the internal pilot which included the number of recruiting sites, the number of recruited participants, the delivery of the first STEPWISE intervention and rates of retention at 3 months.

### Protocol changes

The trial is largely being conducted as it was originally envisaged. Prior to starting the trial, the method of calculating the primary outcome (weight) was altered from absolute weight to change in weight (kg) following advice received from the first TSC meeting. A small number of other modifications have been made, some of which were a result of the Intervention Development Study whilst others relate to the group-based intervention (e.g. sites were encouraged to recruit in waves not too far ahead of a scheduled course).

In conclusion, the STEPWISE trial will provide evidence for the clinical and cost-effectiveness of a group-based structured education programme designed to help people with schizophrenia, schizoaffective disorder or first-episode psychosis to lose weight or attenuate weight gain. If the intervention is successful, the design of the intervention and trial will allow a rapid dissemination within the NHS.

### Trial status

Participant recruitment finished on 31 March 2016 (*n* = 414 recruited) and the trial is in follow-up.

## Additional file

Additional file 1: Table S1. STEPWISE foundation and booster session curriculum. (PDF 9 kb)

## Abbreviations

CEACs: Cost-effectiveness acceptability curves
CMHT: Community mental health team
CONSORT: Consolidated Standards of Reporting Trials
DESMOND: Diabetes Education and Self-management for Ongoing and Newly Diagnosed
DOT: DESMOND Observational Tool
EQ-5D-5L: EuroQol five dimensions, five levels questionnaire
GEE: Generalised estimating equation model
ICD-10: *International Classification of Diseases (version 10)*
ICERs: Incremental cost-effectiveness ratios
MRC: Medical Research Council
NPT: Normalisation Process Theory
OPCRIT: OPerational CRITeria checklist for psychotic illness and affective illness
QALY: Quality-adjusted life year
RCT: Randomised controlled trial
SF-36: 36-Item Short Form Survey (RAND Health)
STEPWISE: STructured lifestyle Education for People WIth SchizophrEnia
