# Supplementary material for: STEPWISE – STructured lifestyle Education for People WIth SchizophrEnia: a study protocol for a randomised controlled trial
Source: Trials. 2016 Sep 29;17:475. doi: 10.1186/s13063-016-1572-1 (PMC5041320; doi:10.1186/s13063-016-1572-1)
Supplement: Additional file 1: Table S1. — STEPWISE foundation and booster session curriculum. (PDF 9 kb) [file 13063_2016_1572_MOESM1_ESM.pdf]

Additional file 1: STEPWISE Foundation and Booster Session Curriculum

	Foundation course				Booster
	Session 1	Session 2	Session 3	Session 4	Sessions 1, 2 and 3
**Your story**	Your story	Sharing stories	Sharing stories	Sharing stories	Sharing stories
**Topic sessions**	Taking control of your weight: your medication and your weight	Taking control of your weight: Healthier snacks	Taking control of your weight: Calories and portions	Taking control of your weight: Eating out	1. Cooking healthy meals<br>2. Physical activity revisited<br>3. Being in the moment with your food
	Taking control of your weight: healthier drinks	Physical Activity and calories	Sedentary Behaviour	Taking control of your weight: Making changes to food	Keeping it going
**Next steps**	Next steps	Next steps	Next steps	Making a plan	Next steps
**Supporting tool**	Weighing scales & water bottle	Pedometer	Cookery book Food scales	Tape measure	1. Mixed herbs, spices, stock cubes<br>2. Theraband<br>3. Fridge magnet, calendar
